# Autism Spectrum Disorder in the Educational Context: A Detailed Case Report

**DOI:** 10.7759/cureus.88149

**Published:** 2025-07-17

**Authors:** Viliam Tomljenovic

**Affiliations:** 1 Department of School and Adolescent Medicine, Teaching Institute for Public Health "Dr. Andrija Štampar", Zagreb, HRV

**Keywords:** autism spectrum disorder, behavioral interventions, inclusion, multidisciplinary care approach, neurodevelopmental disorders

## Abstract

Autism spectrum disorder (ASD) is a complex neurodevelopmental condition characterized by deficits in social communication and interaction, alongside the presence of repetitive and restrictive behaviours. The diagnosis is based on the criteria outlined in the fifth edition of the Diagnostic and Statistical Manual of Mental Disorders (DSM-5) and requires a multidisciplinary and longitudinal clinical approach. Early symptom identification and timely intervention significantly influence treatment outcomes and the child’s functional capacity. This case report presents a male child with pronounced developmental, communicative, and adaptive difficulties evident from early childhood. He demonstrated symptomatology indicative of ASD in at least three categories of the diagnostic manual. Throughout a multi-phased diagnostic and therapeutic process conducted over several years, the child exhibited difficulties in the processing of sensory stimuli, language deficits, emotional dysregulation, and limited adaptability to new social environments. Despite participation in a customized educational program and implementation of therapeutic interventions (integrative sensory therapy, speech-language pathology, psychopharmacology), the child's schooling was marked by frequent behavioral decompensation and a strained collaboration between parents and the educational system. This report highlights systemic deficiencies in the care infrastructure for children with ASD, particularly concerning human resource limitations, inconsistent institutional support, and inadequate coordination between healthcare and educational services. The lack of clear jurisdiction and procedural guidelines hinders the establishment of optimal educational models. A comprehensive reform is imperative, aiming to develop an integrated, individualized, and professionally grounded support system. ASD requires a comprehensive, coordinated, and individualized therapeutic and educational approach, with continuous assessment of treatment efficacy. The report underscores the urgent need for systemic changes in the care and education of children with developmental disorders to improve their overall quality of life.

## Introduction

The concept of autism was first introduced in 1943, when psychiatrist Leo Kanner conducted a clinical study describing specific behavioral patterns in children who exhibited pronounced difficulties in establishing social connections with peers, alongside heightened sensitivity to changes in their social environment [1]. Since then, the reported prevalence of the condition-initially termed autism-has steadily increased. The study by Shaw et al. [2] found that in 2022, the prevalence of autism spectrum disorder (ASD) among eight-year-old children was 32.2 cases per 1,000 (roughly 1 in 31 children) with a male-to-female ratio of 4.5:1. The observed increase in ASD prevalence is largely attributed to substantial improvements in the development and application of diagnostic criteria [2]. The fifth edition of the Diagnostic and Statistical Manual of Mental Disorders (DSM-5) outlines five principal domains used by clinicians to confirm or rule out a diagnosis of ASD [3]. In addition to deficits in social interactions and verbal and nonverbal patterns of response, these also involve the exclusion of other potential causes with similar symptomatology, such as global developmental delay or intellectual disabilities.

In DSM-5, ASD serves as an umbrella diagnosis that encompasses several conditions previously categorized as distinct, including autistic disorder, Asperger’s syndrome, childhood disintegrative disorder, and pervasive developmental disorder not otherwise specified [1]. The diagnosis of ASD is established through consensus by an interdisciplinary team and must adhere strictly to the criteria defined in the DSM-5. Furthermore, various standardized tools, such as the ADOS (Autism Diagnostic Observation Schedule) and TROG (Test for Reception of Grammar), are commonly used to assess the communication and cognitive domains in individuals with ASD, providing valuable insights into their diagnostic and developmental profiles [4].

The therapeutic approach to ASD is multifaceted and typically long-term, involving a combination of intensive behavioral therapy and adjunctive pharmacological treatment when indicated [1]. Behavioral therapy is recommended for a minimum of 15 to 40 hours per week [5]. It is grounded in the principles of applied behaviour analysis (ABA) and focuses on the early identification and replacement of maladaptive behaviors with more functional and socially appropriate alternatives [1]. A comprehensive systematic review conducted in 2014 by the Agency for Healthcare Research and Quality (AHRQ) demonstrated that early behavioral interventions lead to clinically and statistically significant improvements in cognitive and language-adaptive functioning in children with ASD [6].

Pharmacological treatment for ASD should be considered only after a thorough sub-specialist evaluation. Given the potential side effects of available medications, pharmacotherapy is typically reserved for cases where behavioral interventions alone prove insufficient. The U.S. Food and Drug Administration (FDA) has approved two atypical antipsychotics - aripiprazole and risperidone -for the treatment of specific behavioural symptoms associated with ASD [1]. Given that most of the literature references pertain to published studies in the United States, it is important to note that the conceptualization of the diagnosis, therapy, and epidemiological statistics of ASD varies depending on the continent, country, and sociocultural contexts.

A major challenge in the developmental trajectory of children with ASD is their integration into the educational system. Educational challenges faced by these children are multifaceted. Several studies have investigated the relationship between academic achievement and cognitive abilities in children with ASD, often highlighting discrepancies between full-scale IQ (FSIQ) scores and actual academic performance [7,8]. However, there remains a notable gap in research exploring the relationship between core ASD symptoms and social functioning within the school context.

Zaidman-Zait et al. [9] conducted a study examining predictors of academic and social functioning in school-aged children with ASD. Their sample comprised 178 participants, predominantly male (88%). The study identified language skills, nonverbal IQ, behavioral difficulties, and early social interaction deficits as key predictive variables. Using latent profile analysis, children were categorized according to their overall level of educational functioning. Multinomial regression analysis was then employed to assess the extent to which early predictors influenced functional categorization within the educational system. The findings suggest that language abilities and early social interaction difficulties were the only statistically significant predictors. These results underscore the critical role of early behavioral assessment and intervention, particularly during the preschool years, in promoting optimal educational and social development in children with ASD.

One of the key obstacles to creating an inclusive educational environment for children with ASD is the persistent lack of adequate infrastructure, skilled personnel, and effective organizational frameworks within the educational system. This issue was comprehensively examined by Odom et al. [10] in their commentary commemorating the 40th anniversary of the DSM-III's publication. They highlighted enduring obstacles, including inadequate interdisciplinary collaboration among school professionals, insufficient training and preparation of educational and support staff for effectively working with children on the autism spectrum, suboptimal financial compensation that exacerbates staff turnover and perpetuates personnel shortages, and pronounced inconsistencies in educational practices across schools within the same geographical region.

## Case presentation

A 12.5-year-old male was born following a pregnancy complicated by maternal hypertension and delivered via cesarean section. Shortly after birth, he experienced a brief episode of oxygen deprivation, from which he was successfully resuscitated. His Apgar score at birth was 7/10. The child's medical history revealed no significant somatic illnesses. However, until the age of three, he experienced issues related to vesicoureteral reflux, which resolved spontaneously over time. According to parental reports, he began walking at 15 months of age and spoke his first words around his first birthday. Between the ages of three and six, he attended a mainstream preschool. His attendance was discontinued after he began exhibiting severe distress and panic upon being taken to daycare. This included intense crying and resistance, ultimately leading his parents to cease sending him.

As he reached the age of compulsory schooling, an evaluation was conducted to determine his readiness for first grade. During the school medical assessment, the child demonstrated markedly reduced cooperation, rendering several components of the evaluation unfeasible. He was accompanied by his father, who continuously attempted to soothe him throughout the session. Motor assessments revealed clumsiness and immaturity, with noticeable bradykinesia and sluggish adiadochokinesia. The child showed only partial awareness of his body schema, while spatial orientation and analogical reasoning could not be reliably assessed. Significant speech difficulties were observed, including pronounced dyslalia. Due to his limited cooperation, it was not possible to adequately evaluate his graphomotor or perceptual-motor skills.

Emotionally, the boy displayed pronounced anxiety: he refused to enter the examination room independently, remained physically close to his parents, did not respond to questions, and demonstrated emotional dysregulation. His behavior included features of hyperactivity, distractibility, impulsivity, perseveration, and mood instability. Based on the findings, a one-year postponement of school enrollment was recommended. The parents were advised to pursue a comprehensive multidisciplinary evaluation to better understand the child’s difficulties and to identify the most appropriate educational setting. Referrals were made for both neuropediatric and psychiatric assessments.

Following the preschool medical examination, a psychological assessment was conducted. It was found that the child was highly dependent on caregivers for self-care activities. He did not independently perform basic daily tasks such as dressing or feeding, requiring significant assistance, particularly from his mother. Although he had achieved sphincter control, he exhibited persistent fear of using the toilet and continued to request diapers. During the evaluation, he appeared anxious, tearful, and fearful, with significant difficulty separating from his parents.

Reports from his previous preschool indicated minimal social interaction with peers and the presence of stereotyped and repetitive behaviors, such as tapping objects and clapping. Additionally, he demonstrated heightened sensitivity to auditory stimuli. While he showed situational understanding of conversational flow, his receptive language abilities were significantly impaired. In spontaneous communication, he exhibited echolalia and frequently responded inappropriately, producing structurally and logically disorganized utterances. The psychological assessment concluded with recommendations for further diagnostic clarification and a continued delay in school enrollment.

Subsequently, the boy underwent a speech and language assessment. During this evaluation, he was verbally responsive but demonstrated marked agrammatism, frequent digressions, and repetitive vocalizations. Episodes of crying and visible anxiety were noted throughout the session. His expressive vocabulary was limited, and auditory discrimination of words and non-words appeared immature for his age. Although he was able to count in rote sequences, he had not developed an understanding of numerical concepts or quantitative relationships. He was unable to name or differentiate colors based on verbal prompts. Dyslalia was again noted in his speech. His comprehension of grammatical structures was moderately impaired, as indicated by performance on the TROG-2 test. The assessment of general speech comprehension was disrupted due to emotional distress; the boy became tearful, reacted strongly, and insisted on returning home.

Following the speech therapy assessment, the boy underwent a specialist evaluation by a child and adolescent psychiatrist. During the psychiatric assessment, contact with the boy was successfully established, though it was evident that his understanding of conversational flow was impaired. After a brief interaction with the psychiatrist, the boy began to exhibit symptoms of anxiety. Stereotypical behaviors and vocalizations were observed as his anxiety heightened. His attention was described as distractible, and he displayed significant difficulty maintaining focus. After the evaluation, an initial diagnosis of pervasive developmental disorder was made. Table 1 presents a concise description of the symptoms and signs observed in the boy during the multidisciplinary evaluation, as well as during the examination and assessment conducted by the school physician. 

**Table 1:** Symptoms and Signs Observed in the Boy During Multidisciplinary Evaluation and Routine Pre-School Medical Assessment For improved clarity, the author provides a descriptive overview of the symptoms and signs observed in the patient during the periods of multidisciplinary assessment and preschool evaluation, presented in table format. The table compares these observations across both evaluation phases, highlighting key challenges in communication, behavior, and emotional regulation

Symptoms and Signs	Multidisciplinary Diagnostic Evaluation	Routine Preschool Observation and Assessment
Social Communication	Persistent challenges in initiating and sustaining social communication. The child seldom initiated interaction and showed minimal responsiveness to social cues. Reciprocal exchanges were notably absent, with little engagement in shared affect or joint attention.	Verbal and nonverbal social interactions were minimal. The child did not spontaneously engage with peers or adults and showed little interest in reciprocal communication, either verbal or gestural.
Motor Behavior	Stereotyped motor behaviors—particularly hand flapping and foot tapping—were observed frequently. These appeared self-soothing and were triggered by both emotional arousal and sensory input.	Repetitive movements such as body rocking, hand flapping, and finger flicking were consistent and non-contextual, occurring both during structured tasks and free play.
Adaptability and Emotional Regulation	The child exhibited marked emotional dysregulation in response to changes in the environment. He cried frequently, resisted examiner directives, and showed significant distress with minor transitions or unfamiliar stimuli.	Engagement was hampered by low frustration tolerance and resistance to routine changes. The child struggled with transitions between activities and required caregiver support to remain in the classroom setting.
Nonverbal Communication	Eye contact was fleeting or absent. The child did not use facial expressions or gestures (e.g., pointing, nodding) to communicate intentions or emotional states. These deficits disrupted interactive flow.	Nonverbal communication was limited. The child relied heavily on physical gestures (e.g., pulling an adult’s hand) but did not use socially meaningful nonverbal cues. Facial expressions were incongruent or absent during interaction.
Sensory Sensitivities	Pronounced auditory hypersensitivity. Loud or unexpected noises (e.g., door slamming, raised voices) triggered immediate anxiety responses such as covering ears and becoming visibly tense or tearful.	The child showed heightened sensitivity to environmental stimuli. He avoided noisy areas, became agitated with loud toys, and required a quieter setting to participate meaningfully in group activities.
Fixation/Restricted Interests	Not prominently observed in this phase but emerged briefly during unstructured moments with repeated engagement in sensory-seeking behaviors.	Strong fixation on specific objects (e.g., spinning toys, textured items). The child became preoccupied and was difficult to redirect, interfering with task engagement and peer interaction.
Separation Anxiety	Demonstrated distress when transitioning between rooms or separating from caregivers, but was somewhat manageable with familiar adult presence.	Marked separation anxiety. The child became tearful, clung to caregivers, and refused to enter the classroom alone, significantly limiting independent observation.

A subsequent neuropediatric evaluation confirmed the psychiatric findings, revealing no significant neurological abnormalities. Given the boy's limited cooperation and the need for further exploration, it was recommended that he be included in a more comprehensive neuropediatric program at a day hospital. This program would include further diagnostic testing, such as electroencephalography (EEG) and visual evoked potentials.

At the beginning of the following year, the boy was re-evaluated by a child and adolescent psychiatrist at a different medical facility. The conclusions of this assessment were consistent with those from the initial psychiatric evaluation. Considering the increasing intensity of his symptoms, particularly those suggestive of ASD, a multidisciplinary evaluation was recommended. However, this recommended evaluation had never been done.

Following the one-year delay in school enrollment, a new assessment was conducted by the school doctor. The parents provided an opinion and report from the daycare that the boy had attended previously. The findings of this assessment indicated some improvement in the boy’s social and communication skills, as well as enhanced emotional regulation and better attention control. Despite these advances, the boy continued to systematically refuse participation in most scheduled activities. Based on the daycare’s feedback and the conclusions drawn from the multidisciplinary assessments, which included speech therapy, psychological, psychiatric, and neuropediatric evaluations, the recommendation was made for the boy’s enrollment in first grade under special circumstances. With the parents' consent, assistance from a teaching assistant was requested, and it was proposed that the boy’s education follow a specialized program with individualized approaches within a separate classroom for children with behavioral difficulties.

Upon entering this new educational setting, the boy experienced significant difficulties adapting. Due to his attachment to his parents, he struggled greatly with separation and adjusting to the school environment. During episodes of separation anxiety, he would verbally express his distress at being in the school setting. His sensory hypersensitivity became more pronounced, with exaggerated reactions to acoustic stimuli from the environment. He would scream, cry, and request his parents, vocalizing a desire to leave the school. Over time, disturbances in the classroom environment became more frequent and intense, with the boy occasionally throwing objects off his desk. In response to the escalation of symptoms, his parents enrolled him in a sensory integration program, where a therapeutic dog was introduced to assist with his treatment.

An initial assessment of the sensory integration program described the boy as constantly seeking support from his parents and displaying significant dependence in performing daily tasks. Despite this, he demonstrated communication skills and an interest in his environment. Initially, he was highly active, engaging in behaviors such as running, jumping, and carrying balls. His behavior exhibited stereotypies, and he remained in a heightened state of arousal. During play, he displayed limited ideation and lacked creativity when interacting with objects. Socio-emotionally, he exhibited significantly lower self-confidence and a persistent need for encouragement. He displayed fear in interactions with both peers and the therapy dog, initially hesitating to engage. However, with individualized support, he gradually began to interact more effectively.

According to the parents, sensory integration therapy produced some positive outcomes. The boy exhibited reduced sensitivity to auditory stimuli; however, challenges related to school adaptation persisted. As he was included in a partially integrated educational model within a special classroom setting, he attended subjects such as music, art, and physical education alongside peers from the general education program. Participation in these activities remained significantly challenging, largely due to his fear of using stairs and heightened sensitivity to auditory input from the regular classroom environment. Although sensory therapy partially mitigated these issues, it was only by the end of the lower grades in primary school that the boy was able to attend general education classes more consistently.

In the third grade, the boy underwent new speech and psychological evaluations. The findings from the speech therapy assessment mirrored those obtained during the initial pre-school evaluation. Based on clinical observations, the use of the ADOS (Autism Diagnostic Observation Schedule) protocol was recommended for further diagnostic clarity. The psychological assessment reiterated earlier conclusions and highlighted ongoing difficulties with sensory processing, social communication, and attentional regulation, all consistent with functioning within the spectrum of mild intellectual disability. Continued support through the presence of a teaching assistant and maintenance of the existing educational framework were advised.

Throughout his schooling, the boy encountered significant adaptation difficulties, which led to frequent changes in teaching assistants. Reports from school personnel documented several incidents during which the boy's behavioral decompensations escalated into physical outbursts directed at assistants. The most pronounced difficulties began with the transition into the upper grades of primary education. Although he continued in a partially integrated classroom setting, his time spent in class was increasingly limited. After the first lesson of the day, he would often voice dissatisfaction, stand up, throw objects, and express a desire to leave and return home. According to the educational rehabilitation specialist, the teaching process was regularly disrupted by his behavioral episodes, and lessons were frequently interrupted.

In response to the worsening of symptoms, the boy was brought to regular follow-up appointments with a pediatric and adolescent psychiatrist. Due to the severity of the behavioral decompensations, a pharmacological intervention was initiated. The prescribed regimen included risperidone and quetiapine, with as-needed administration of diazepam. The dosage was gradually titrated over several appointments. Although the boy’s father reported noticeable behavioral improvement, school staff continued to observe behavioral outbursts and classroom disruptions with unchanged frequency and intensity. This persistent discrepancy in perspectives - largely grounded in the subjective judgments of both the parents and the school staff - became a central source of tension, ultimately straining their relationship.

In light of the ongoing challenges, school authorities initiated procedures to organize a multidisciplinary re-evaluation of the student. The aim was to assess whether a change in educational placement-either a fully integrated special classroom or transfer to a specialized institution for children with ASDs-might be warranted. The parents strongly opposed this proposal, voicing concerns that such a transition would exacerbate the boy’s psychological and emotional distress and potentially deteriorate his physical health. The attending child and adolescent psychiatrist supported the continuation of the current educational model. Although the school doctor also recommended a team-based reassessment, the parents, feeling alienated due to the escalating conflict with the school, remained unreceptive to this recommendation.

The school formally reported the situation to the relevant social welfare center and proposed initiating procedures to alter the boy’s current educational framework without parental consent. It was anticipated that the parents would file an appeal in response to this proposal. The school physician was unable to issue an opinion regarding the proposed change in educational modality, as a comprehensive team re-evaluation had not yet been conducted, and no formal conclusion had been reached indicating a need to modify the existing educational framework. Additionally, the most recent findings from the attending child and adolescent psychiatrist recommended the continuation of the current educational model. 

**Figure 1:**
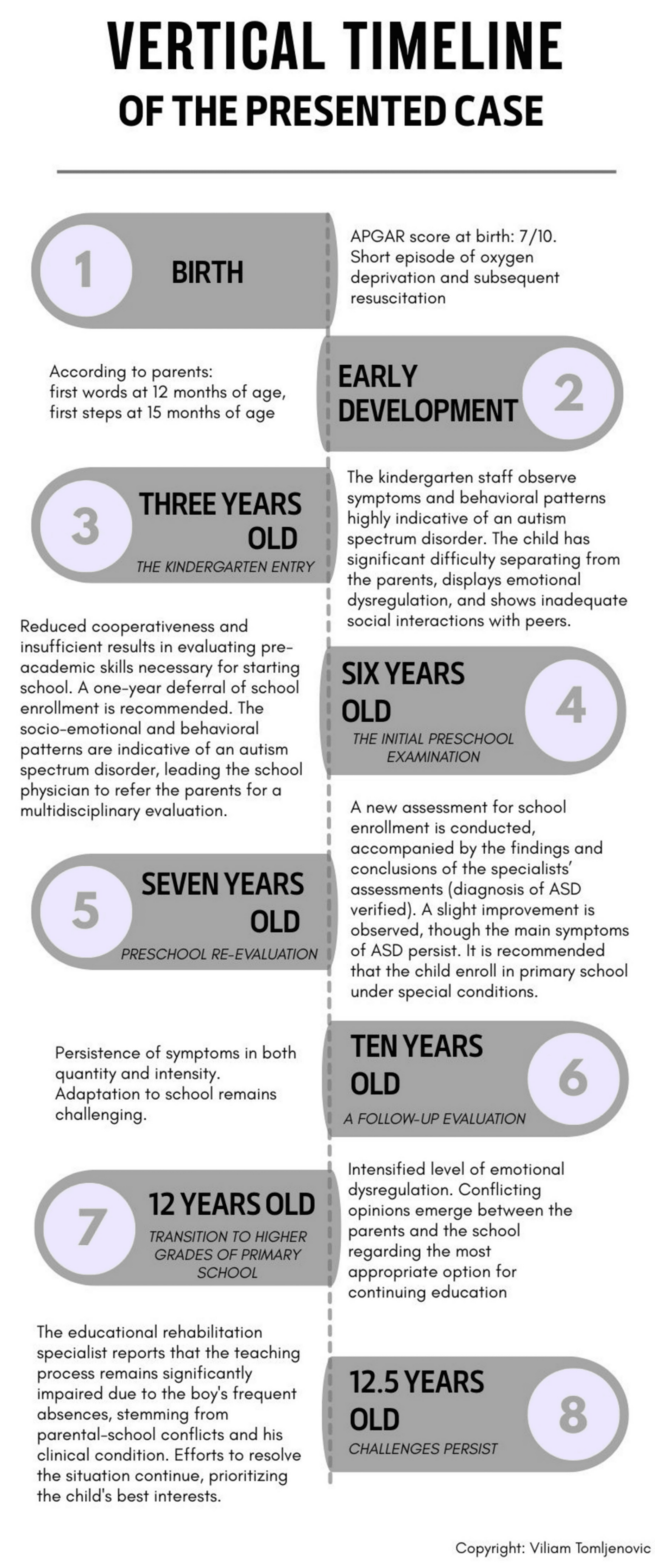
Vertical Timeline of Key Milestones in Psychophysical and Socio-Emotional Development From Birth to Case Resolution The figure presents a chronological sequence of events related to the development of symptoms, the complex and prolonged multidisciplinary evaluation, and the challenges of educational inclusion

According to documentation from the educational rehabilitation specialist, the teaching process remains significantly impaired. The boy continues to be frequently absent from school as a result of both the ongoing conflict between his parents and the school administration, as well as the nature of his clinical condition. The situation remains unresolved. A brief overview of key events is presented in Figure 1.

Continued efforts are being made to reach a mutually acceptable compromise, with primary emphasis placed on safeguarding the best interests of the child. Table 2 provides a structured summary of the clinical features, multidisciplinary assessment findings, and educational interventions described throughout the case presentation.

**Table 2:** Summary of Clinical Features, Multidisciplinary Findings, and Educational Interventions in a 12.5-Year-Old Boy With Developmental and Behavioral Challenges This table provides a structured summary of the child’s clinical trajectory, multidisciplinary evaluation findings, therapeutic interventions, and evolving educational needs, with emphasis on key behavioral, cognitive, emotional, and environmental factors

Domain	Findings/Observations
Perinatal History	Cesarean section due to maternal hypertension; brief neonatal oxygen deprivation; Apgar score 7/10
Early Development	Delayed walking (15 months); first words by 12 months; early vesicoureteral reflux (resolved by age 3)
Preschool History	Mainstream preschool; discontinued due to panic and separation anxiety
Preschool Evaluation	Poor cooperation, motor immaturity, bradykinesia, speech deficits (dyslalia), emotional dysregulation
Psychological Assessment	Dependency in self-care, persistent diaper use, high anxiety, minimal peer interaction, echolalia, sensory sensitivity
Speech & Language	Agrammatism, limited vocabulary, impaired auditory discrimination, dyslalia, TROG-2 showed moderate grammatical deficits
Psychiatric Evaluation	Impaired conversational flow, stereotypies, distractibility, anxiety; initial diagnosis: *pervasive* *developmental disorder*
Neuropediatric Evaluation	Confirmed psychiatric findings; no neurological abnormalities; recommended further day hospital diagnostic testing
Educational Interventions	1-year enrollment delay; placement in partially integrated classroom; support of teaching assistant
Sensory Integration Therapy	Managed to reduce auditory sensitivity; improved interaction over time; persistent dependence noted
Ongoing Challenges	Classroom behavioral outbursts, difficulties with transitions, fear of stairs, disrupted learning environment
3rd Grade Re-evaluation	Persistent speech-language and cognitive deficits; functioning within mild intellectual disability range
Pharmacological Treatment	Risperidone, quetiapine, diazepam PRN; parents reported improvement; school observed continued behavioral difficulties
Parent–School Conflict	Discrepancy in symptom perception; increased tension over placement; parents opposed further institutionalization
Current Status	Frequent absences, unresolved educational placement, pending multidisciplinary reassessment, social services involved

## Discussion

ASD is a complex neurodevelopmental condition that necessitates early identification, accurate diagnostic evaluation, and a sustained, multimodal therapeutic strategy [1]. Contemporary diagnostic criteria, as outlined in the DSM-5 classification [3], facilitate earlier and more precise recognition of ASD symptomatology, with interdisciplinary collaboration among professionals being essential to the diagnostic and intervention process. While symptoms typically arise in early childhood, the full clinical presentation often becomes more apparent in contexts that place increasing demands on social functioning [1]. The prevalence of ASD is estimated at approximately 1 in 31 children [2].

This case report generally aligns with current diagnostic and therapeutic protocols. The diagnosis, established according to DSM-5 criteria [3], was based on the presence of three criterion groups from categories A and B, early symptom onset, functional impairment, and the absence of an alternative etiological explanation. The therapeutic approach initially involved sensory interventions, followed by the introduction of psychopharmacological treatment options. Risperidone therapy is consistent with current clinical recommendations and regulatory approvals [1]. Despite comprehensive diagnostic evaluation and the child's early involvement in sensory habilitation, standard behavioral therapy-implemented in accordance with recommended intensity and duration guidelines [5], was not adequately administered. 

The educational challenges faced by children with ASD are multifaceted and extend well beyond academic performance, with a strong emphasis on difficulties related to social inclusion [10]. The success of educational inclusion is closely linked to the child’s language competence and the quality of early social experiences [9]. This is evident in the case of the boy presented in this report. The symptomatology of his disorder significantly interfered with his ability to adapt appropriately to the school environment. He struggled to navigate the school setting and failed to establish constructive social relationships, while the educational process has been persistently impaired from the outset.

Although significant progress has been made in the early identification and educational inclusion of children with ASD, there remain substantial gaps in the practical implementation of support systems [10]. Drawing on published literature, practical experience, and the context of the presented case, it appears that current educational and healthcare frameworks remain insufficiently adapted to meet the complex and diverse needs of this population. The shortage of adequately trained professionals, variability in service quality, and the absence of coordinated inter-institutional collaboration highlight the need for comprehensive systemic reform.

To address these challenges, it is imperative to develop an integrated, cross-sectoral model of support that enables a highly individualized approach and fosters optimal developmental outcomes for children with ASD.

## Conclusions

This case highlights the complex interplay between neurodevelopmental difficulties, emotional regulation challenges, and environmental factors in shaping a child’s educational trajectory. Despite early recognition of developmental concerns and multiple professional evaluations, the implementation of recommended interventions remained fragmented and inconsistent. The absence of coordinated multidisciplinary follow-up and clearly defined roles among healthcare and educational professionals contributed to ongoing challenges in managing the child’s needs. Although the child received an early diagnosis and participated in some therapeutic interventions, the absence of a structured, standardized behavioral treatment program likely contributed to the ongoing difficulties in his educational functioning. Furthermore, discrepancies between parental and school perspectives, often grounded in subjective judgments, led to escalating conflict, ultimately hindering collaborative decision-making in the child’s best interest. Notably, the Autism Diagnostic Observation Schedule (ADOS), despite being recommended, was never administered, which weakens the diagnostic rigor and limits the objectivity of clinical conclusions.

Strategic investments of both financial and human resources are essential to restructure the current system in a manner that enables a more individualized and child-centered approach. One of the most pressing concerns lies in the insufficient staffing capacity and the evident shortage of qualified professionals who can provide continuous and specialized support to this vulnerable population. These professionals must be adequately educated and trained to work with children who present with specific medical and developmental profiles, offering guidance, encouragement, and interventions that support each child’s optimal developmental trajectory. This segment of reform particularly pertains to the system of teaching assistants, who frequently receive inadequate compensation and often lack the specialized training required to support children with complex and sensitive conditions. The systemic issue further extends into the healthcare domain. The field of school and adolescent medicine is limited by its non-curative mandate, and children under the supervision of outpatient services frequently fall into a gap between the primary pediatrician from whom they transition, the general practitioner who provides family-based care, and the school physician responsible for preventive examinations and issuing educational recommendations and certifications. The lack of clearly defined roles and responsibilities within this continuum of care constrains healthcare professionals in their ability to provide coordinated, comprehensive, and effective support for the child.
